# Army Dental Corps

**Published:** 1905-01-15

**Authors:** J. S. Marshall


### ARMY DENTAL CORPS.

BY J. S. MARSHALL, M.D., D.D.S.

I desire to invite your attention to certain well-established scientific facts in relation to the prevalence of dental diseases among civilized nations, and also certain other facts gained from observation and experience in military dental practice relative to the great need of the services of dental surgeons in the United States Army. I also believe, judging from reports coming from British and German military authorities, there is equal need of such services in those armies, and I have no doubt, if the facts were known, similar need would be found to exist in all the armies of the civilized world.

It is a fact generally admitted by dental pathologists and practitioners that dental caries and its sequelæ are the most common diseases of the human race, and that these diseases are constantly increasing in prevalence, particularly among civilized nations; and as further proof of this statement the following statistics are presented:

The prevalence of dental caries among American grammar-school children was computed by Ottofy from an examination of 14,644 teeth. Caries was present in 27.33 percent in males and 32.67 percent in females. In an examination of the naval apprentices of the British training ship "Exmouth" made at about the same period, the average age of the boys being about fourteen years, it was found that 76 percent of them had carious teeth. In a more recent examination of the teeth of the school children of Northern Germany, conducted by Dr. Greve of Lubeck, the ages of the children being between twelve and fifteen years, it was found that 94.5 percent of them had dental caries in its various stages of development.

The statistics of the United States army dental corps show that dental diseases are as prevalent among the officers and enlisted men of our army as among individuals in civil life of the same social class, while among the troops who have served in tropical and semi-tropical climates these diseases are much more prevalent.

The Surgeon-general's report for 1903 shows that the percentage of dental diseases for troops serving only in the United States was 42.85. For those serving in the Philippine Islands the percentage was 61.12, or 18.27 percent higher than for the troops who served only in the United States; while for the troops who served in Cuba and Porto Rico the percentage was 64.02, or 21.17 percent higher than for the troops serving in the United States. The increased percentage of dental diseases among the troops who served in Cuba and Porto Rico over those serving in the Philippine Islands is due to the fact that these diseases are generally more prevalent among the native troops of Porto Rico than among the white troops.

The records of my office at the General Hospital, Presidio of San Francisco, show that from October 1, 1901, to July 1, 1904, 4,533 cases have been examined and treated for dental and oral diseases, and out of this number only one person has been found who was absolutely free from dental caries; this was a young lieutenant just graduated from West Point military academy.

In an examination conducted by two of my assistants of two regiments of infantry recently returned from the Philippines, it was found that in one regiment 87.62 percent and in the other 93.46 percent of the men examined wer in need of immediate dental treatment.

The statistics for the first-mentioned regiment are as follows:

| | |
|---|---|
| Total No. enlisted Men | 738 |
| No. absent for various reasons | 27 |
| No. enlisted men examined | 711 |
| No. not needing dental treatment | 77 |
| No. needing dental treatment | 623 |
| Percent of regiment needing treatment | 87.62 |

DISEASES.

| | |
|---|---|
| No. of teeth with dental caries | 2280 |
| So badly diseased as to require extraction | 321 |
| Can be saved by appropriate treatment | 1959 |
| Men needing immediate treatment for pulpitis and dento-alveolar abscess | 277 |
| Cases of salivary deposits | 182 |
| Cases of gingivitis | 42 |
| Cases of pyorrhea alveolaris | 4 |
| Cases needing artifical dentures | 14 |

The statistics for the other regiment are as follows:

| | |
|---|---|
| Total No. enlisted men in the regiment | 780 |
| No. absent for various reasons | 15 |
| No. enlisted men examined | 765 |
| No. not needing dental treatment | 21 |
| No. needing dental treatment | 744 |
| Percent of regiment needing treatment | 93.46 |

DISEASES.

| | |
|---|---|
| No. of teeth with dental caries | 3565 |
| So badly diseased as to require extraction | 197 |
| Can be saved by appropriate treatment | 3221 |
| Men needing immediate treatment for pulpitis and dento-alveolar abscess | 97 |
| Cases of salivary deposits | 196 |
| Cases of gingivitis | 60 |
| Cases of pyorrhea alveolaris | 10 |
| Cases needing artificial dentures | 18 |

The Surgeon-general's report above referred to shows that during the calendar year 1902 there were 16,161 officers and enlisted men treated for dental and oral diseases, this number being exactly 20 percent of the mean strength of the army for that year, and that 49,483 operations were performed. These operations were largely of an emergency nature and necessary to fit the men to perform their military duties. The average number of operations performed for each person was 3.06.

During the Boer war the British military authorities found it necessary to take cognizance of the fact that their troops in the Transvaal were suffering greatly for the need of the services of skilled dental surgeons. This need was recognized by the government, and one dental surgeon with twelve assistants—undergraduate students from the

London Dental Hospital—were sent out to meet this need, inadequately of course, if I may be permitted to judge from my own experience in the United States army.

More recently the British government has regularly appointed eight dental surgeons for duty with the troops of the United Kingdom, but medical officers who are familiar with the needs of the service in this direction have called attention to the fact that this number is entirely inadequate, and that if the desired results are to be obtained a much larger number must be provided.

The German government, through Dr. Richter, a staff surgeon of the German army, has been investigating the question of the need of dental surgeons in the German military service, and he reports that upon the examination of 1,000 men of a Saxon regiment he found only 61 who had thoroughly sound teeth, or in other words 93.9 percent of the men were in need of dental treatment. Of this number there was an average per capita of nearly two teeth missing and more than four decayed. Of the decayed teeth less than one-half could have been saved by proper treatment, the remainder requiring extraction. In another regiment Dr. Richter found that the number of teeth extracted yearly was about 2,300, while 2,100 fillings had been placed in decayed teeth and fifty sets of artificial teeth had been supplied. It will be noticed that these statistics are so near to those of the United States army as to be practically the same, thus proving conclusively that dental and oral diseases are about equally prevalent in all civilized nations.

As a result of the investigations of Dr. Richter the general staff of the German army has authorized the issue of instructions to the entire German army in relation to the hygiene of the mouth. Lectures upon the various branches of the subject have been arranged for, attendance upon which is compulsory, and non-commissioned officers are required to see that all prescribed regulations relating to the care of the teeth are rigorously enforced. The medical officers of the German army are hoping that the inquiries of Dr. Richter will lead to the establishment of a corps of dental surgeons.

Similar recommendations were made by myself to the Surgeon-general of the United States army more than two years ago.

When the facts are taken into consideration that the officers and enlisted men entering military life are in the flower of their young manhood and that the military medical examiners exclude all but the physically perfect, these statistics in relation to the condition of the teeth of the soldiers is somewhat alarming, and the question that very naturally arises in our minds is, What is to be the outcome of this rapid deterioration of the teeth? Good teeth, or at least serviceable teeth, are exceedingly necessary as a means of maintaining the general health of the individual soldier and consequently of the highest efficiency to an army, particularly when campaigning in the tropics, where the conditions of climate are so enervating and debilitating to the general system, and the necessary changes in the habits of life to meet the new environments are not conducive to the highest physical development and vigor. Resistance to disease under these conditions is greatly lessened, and the individual is consequently predisposed to a certain class of diseases, among which are dental caries, pulpitis, pericementitis, dento-alveolar abscess, pyorrhea alveolaris, necrosis of the jaws, inflammatory and ulcerative conditions of the gums, the oral mucous membrane, the throat and tongue; while various general affections are superinduced as a result of the diseased condition of the teeth and oral cavity or the crippling of the function of mastication by the loss of the teeth, such as indigestion, dyspepsia, gastritis, enteritis, colitis, and numerous nervous affections. The remedy for this alarming condition of dental and oral diseases lies in teaching the soldier the value of oral hygiene, and in furnishing the army with an adequate number of dental surgeons to properly care for the dental diseases of the troops in garrison and in the field. The number of dental surgeons in the United States army and in the British army are entirely inadequate for the everpresent and increasing needs in this direction.—*Cosmos.*

